# Ruptured Left Ovarian Teratoma Presenting as an Irreducible Right Inguinal Hernia

**DOI:** 10.1055/s-0041-1736667

**Published:** 2022-02-01

**Authors:** Mohamed Taher Mithi, Swanit Hemant Deshpande, Taher Abbas Mithi, Swarika Hemant Deshpande

**Affiliations:** 1 Deprartment of Oncosurgery, GCRI, Ahmedabad, Gujarat, India; 2 Department of General Surgery, BYL Nair Charitable Hospital and TN Medical College, Mumbai, Maharashtra, India; 3 Department of General Surgery, Saifee Hospital, Mumbai, Maharashtra, India; 4 Department of Obstetrics and Gynecology, KEM Hospital and Seth GS Medical College, Mumbai, Maharashtra, India

**Keywords:** ovarian teratoma herniation, inguinal hernia, teratoma, dermoid, general surgery, onco-surgery, gynecology

## Abstract

Teratomas of the ovary rarely present as inguinal hernias. Teratomas most commonly occur in the gonads or along with midline structures. Although the majority are asymptomatic, complications such as spontaneous rupture are known to occur. We present a previously unreported case of a ruptured ovarian teratoma presenting as an irreducible inguinal hernia. The patient underwent an open exploratory laparotomy with left oophorectomy, and the right inguinal hernia was repaired in the same setting with a separate inguinal incision.

Teratomas most commonly occur in the gonads or along with the midline structures. However, they may also be found at other locations. The most frequent benign tumor in premenopausal women is a mature ovarian teratoma. Teratomas are tumors of germinal origin. They are composed of derivatives of at least two of the three germ cell layers. Ectodermal tissue is almost always present. Bilateral tumors are seen in 8% to 15% of the cases. Most of the patients are asymptomatic. Complications such as adnexal torsion, rupture, malignant transformation, or infection have been reported. The estimated incidence of spontaneous rupture is 0.3 to 2.5%. Aseptic peritonitis can occur in less than 1% of the cases, secondary to the rupture of the tumor. The sebaceous contents irritate the peritoneum, leading to aseptic peritonitis. 1

We present a case of a ruptured left ovarian teratoma with ascites presenting as an irreducible right-sided inguinal hernia.

## Case Presentation (Materials and Methods)

A 36-year-old female presented with a right inguinal swelling for 6 months, which was initially reducible. The swelling had become irreducible, tense, and painful for the past 6 days. There was no history of constipation or any evidence of obstruction. There was no history of malignancy in the family. She was married and had a male child born with a normal vaginal delivery 8 years ago.

On examination, the patient was vitally stable. The patient also had abdominal distension with mild ascites. On clinical examination and history, it was concluded that the patient had a right-sided irreducible inguinal hernia with no signs of obstruction or strangulation with mild ascites.

### Investigations

The patient underwent an ultrasonogram, which showed a right inguinal lesion with multiple septae and echogenic specks. This was suggestive of thick proteinaceous fluid. Moderate ascites with thick internal debris was also noted.

**Fig. 1:**
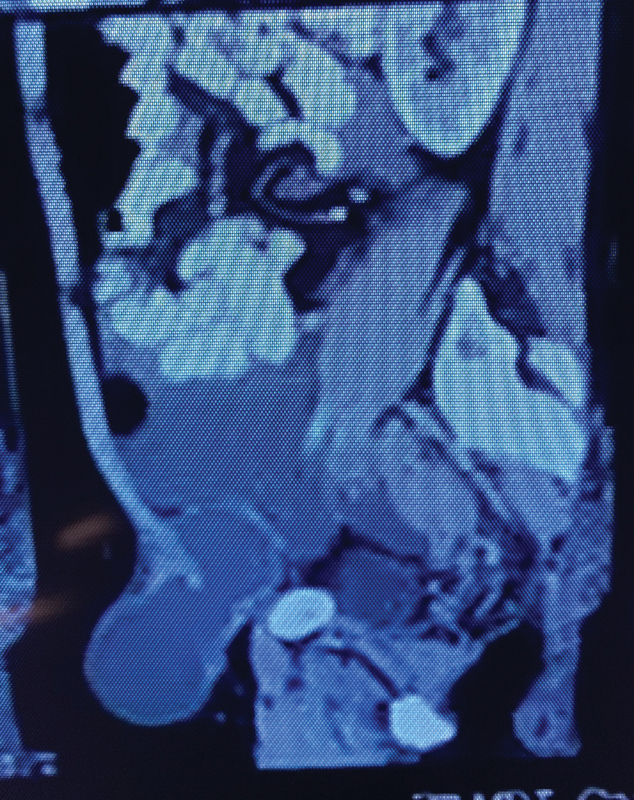
Contrast-enhanced computed tomography (CT) scan image of abdomen and pelvis (saggital section), depicting left ovarian teratoma.

For further evaluation, a contrast-enhanced computed tomography (CT) scan of the abdomen and pelvis was done ( Figs. 1 2 3 ). It showed a large well-circumscribed left ovarian dermoid cyst containing fat fluid-solid component with irregular calcific elements. The right ovary appeared to be normal. The surrounding adnexa was normal in appearance. A right inguinal hernia containing a loculated collection with tiny fat locules and septations along with a mildly thickened and minimally enhancing wall was noted.

**Fig. 2:**
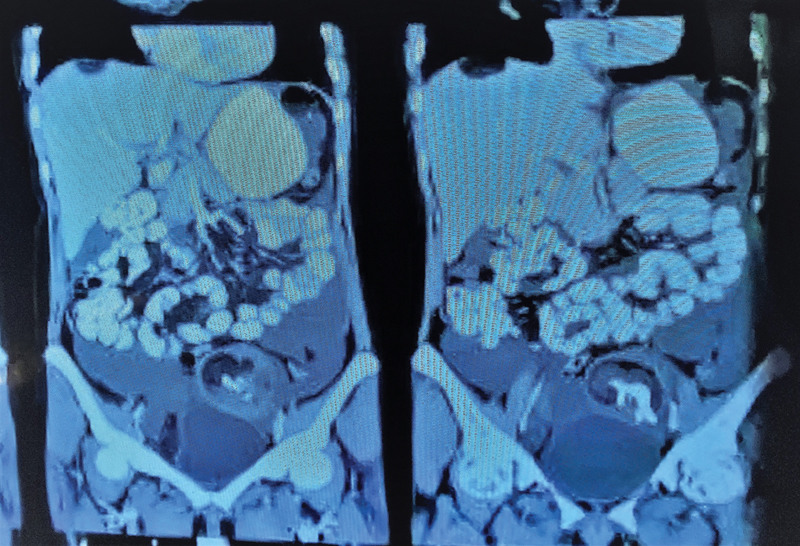
Contrast-enhanced computed tomography (CT) scan image of abdomen and pelvis (coronal section), depicting left ovarian teratoma.

**Fig. 3:**
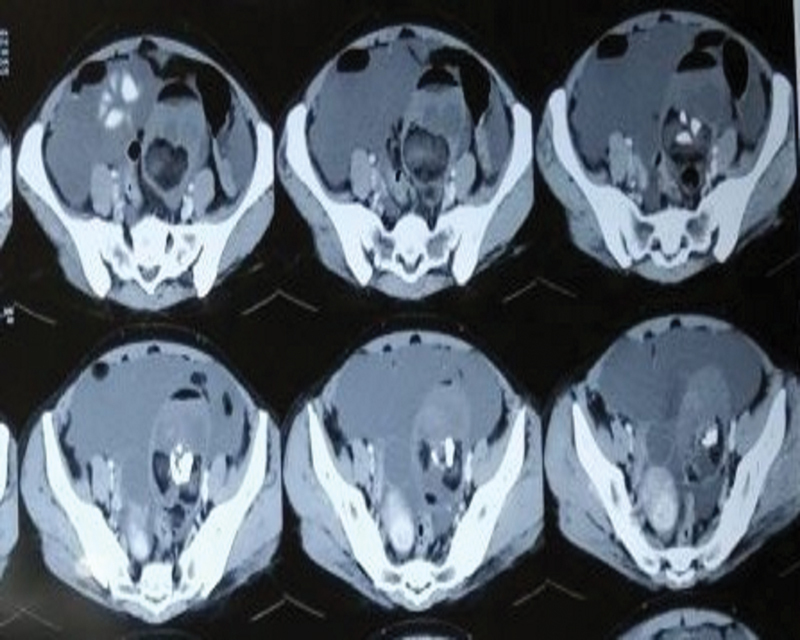
Contrast-enhanced computed tomography (CT) scan image of abdomen and pelvis (axial view) depicting left ovarian teratoma.

Hematological parameters were normal. Tumour markers such as CA 125, HCG, AFP, and LDH were normal.

### Differential Diagnosis

The clinical diagnosis was of an irreducible right inguinal hernia in a patient with ascites. CT scan revealed a possibly ruptured left ovarian teratoma. An inguinal dermoid or a simultaneous right inguinal hernia with enterocele or rectocele were the other differential diagnoses being considered preoperatively.

### Treatment

The decision-making process involved shared decision-making, with the patient being provided with an open as well laparoscopic approach. The patient denied the laparoscopic repair approach due to financial constraints. Open exploratory laparotomy with hernia repair was the surgical procedure decided to be performed. The possibility of left-sided oophorectomy was explained to the patient preoperatively. The patient gave consent for the same.

**Fig. 4:**
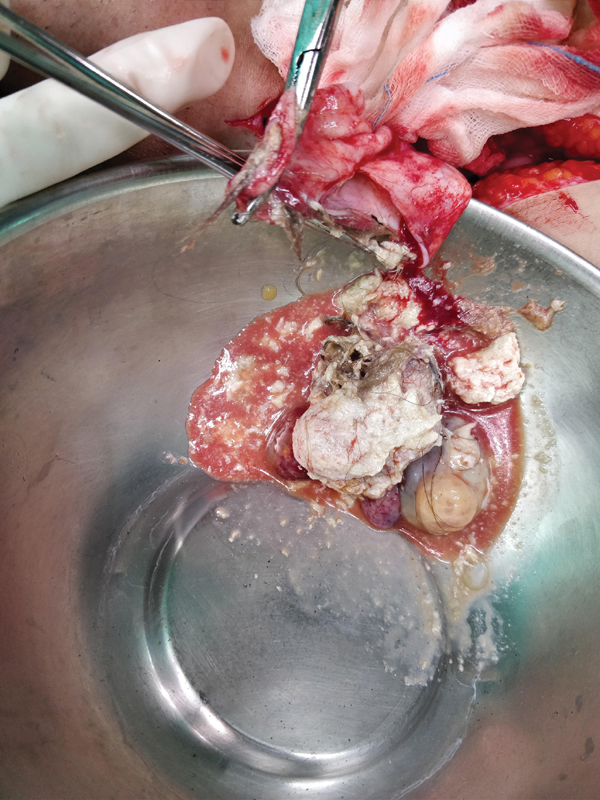
Opened right inguinal sac draining the contents of ruptured ovarian teratoma like hair and paste-like brown material.

**Fig. 5:**
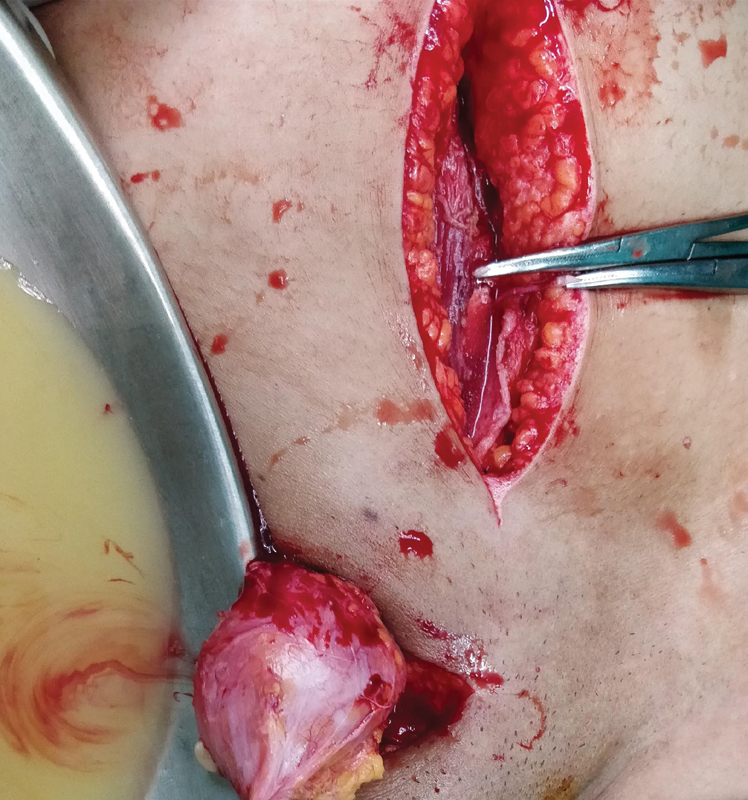
Intraoperative picture depicting the infraumbilical midline incision and the right inguinal incision with hernial sac.

An infraumbilical midline incision was taken ( Figs. 4 and 5 ). On exposure to the peritoneal cavity, 1.5 L of straw-colored fluid was drained. A large ruptured left ovarian cyst was seen which had completely replaced the left ovary. Left oophorectomy was done, and the specimen was sent for histopathological analysis. Uterus, fallopian tube, and right ovary were found to be normal. The bowel was normal. Peritoneal lavage was given, and a tube drain was placed in the pelvis.

The right groin crease incision was then taken for the inguinal hernia. A large direct inguinal hernia was noted. The hernial sac contained paste-like fluid with hair. The sac was excised after draining the fluid. Lichtenstein tension-free hernioplasty was done with a Prolene mesh.

### Outcome and Follow-Up

Histopathology examination confirmed the diagnosis of a mature cystic teratoma (MCT). The ascitic fluid examination was unremarkable.

The patient had an uneventful recovery, and the drain was removed on postoperative day four. The patient was discharged on postoperative day six. The patient was advised to follow-up regularly, and subsequent imaging investigations did not reveal any evidence of recurrence even after 2 years of follow-up.

## Discussion

Teratomas are the most common germ cell tumor of the ovary, with MCT being the most frequent among the various subgroups. 2 Teratomas may present in various ways, including torsion, rupture, or malignant transformation. Serum markers help to distinguish benign from malignant ovarian lesions. It is postulated that increasing estrogen and progesterone levels stimulate the sebaceous gland component of these tumors and in part can explain the increase in size seen in MCTs postpuberty and the arrested growth after menopause. 3 4

With an incidence of 0.3 to 2.5%, spontaneous rupture is a rare presenting feature. 5

In our case, the rupture of a left ovarian MCT presented as an incarcerated inguinal hernia with no evidence of obstruction and minimal peritonitis. Only on the radiological investigation was a preoperative diagnosis of a ruptured ovarian teratoma presumed, and surgical management was altered accordingly to manage the same.

With our review of literature, we could not find a ruptured ovarian teratoma presenting as an irreducible inguinal hernia. Previous case reports have described unruptured ovarian teratoma presenting as inguinal hernia 6 and inguinal dermoid cysts mimicking inguinal hernia. 7

Fertility status may influence the choice between cystectomy or oophorectomy. 8 The Royal College of Obstetrics and Gynecology 9 (RCOG) recommends discussing the possibility of oophorectomy preoperatively with the patient. Ovarian cystectomy may be preferred in younger women unless the patient chooses oophorectomy. In postmenopausal women, oophorectomy should be the standard operation. Oophorectomy should also be considered in perimenopausal women with large ovarian mature cystic teratoma, as there is not much ovarian tissue to conserve 10

Inguinal hernia repair can be attempted laparoscopically or with the open technique. Although the hernia sac contents are sterile, care should be taken to avoid any spillage to reduce secondary infection.

## Conclusions

MCT of the ovary may undergo spontaneous rupture, leading to an aseptic chemical peritonitis.

Although rare, a ruptured MCT of the ovary may present as an inguinal hernia.

The hernia repair can safely be done with a mesh, as the ruptured contents are sterile.
